# Variability in the Content of *Trans*-Resveratrol, *Trans*-ε-Viniferin and R2-Viniferin in Grape Cane of Seven *Vitis vinifera* L. Varieties during a Three-Year Study

**DOI:** 10.3390/molecules22060928

**Published:** 2017-06-03

**Authors:** Jan Tříska, Naděžda Vrchotová, Josef Balík, Ivo Soural, Radek Sotolář

**Affiliations:** 1 Global Change Research Institute, Academy of Sciences of the Czech Republic, v. v. i., Lipová 9, České Budějovice 37005, Czech Republic; vrchotova.n@czechglobe.cz; 2 Faculty of Horticulture, Mendel University in Brno, Valtická 337, Lednice 69144, Czech Republic; josef.balik@mendelu.cz (J.B.); ivo.soural@mendelu.cz (I.S.); radek.sotolar@mendelu.cz (R.S.)

**Keywords:** stilbenes, *Vitis vinifera* L., grape cane, Moravian wine region

## Abstract

Grape canes are a waste product from viticulture that show potential as an industrially extractable source of stilbenes, which are valuable for medical and other purposes. In this work, grape canes collected in three consecutive years (2014–2016) at six different places in South Moravia, Czech Republic were extracted, and the contents of *trans*-resveratrol, *trans*-ε-viniferin, and r2-viniferin were determined by high-performance liquid chromatography. The study included three blue grape varieties of *Vitis vinifera* L. (Cabernet Moravia, Blaufränkisch, and Piwi variety Laurot) and four white grape varieties (Chardonnay, Green Veltliner, Piwi variety Hibernal, and Piwi variety Malverina). From the viewpoint of producing extracts with high stilbenes content, the Hibernal variety is clearly the best. The mean amounts of the stilbenes for this variety at all localities and for all three years were 4.99 g/kg for *trans*-resveratrol, 3.24 g/kg for *trans*-ε-viniferin, and 1.73 g/kg for r2-viniferin. The influence of vintage, locality, and variety on the amounts of stilbenes was studied using PCA analysis. In contrast to expectations, there was no strong impact of locality on stilbenes content. The differences were varietal for most varieties, regardless of the area of cultivation. Laurot and Hibernal varieties did differ significantly in that respect, however, as they exhibited clear dependence on location.

## 1. Introduction

In recent years, increasing attention has been focused on waste products generated in agriculture and in the food industry because such materials can be a source of valuable substances. One group of such substances is the stilbenes. These can be derived from grape canes (a waste material from viticulture), which are very rich in *trans*-resveratrol and *trans*-ε-viniferin. Considering the large amounts of stilbenes found in grape canes (3.45 g of *trans*-resveratrol per kg dry weight (d.w.) of cane and 1.3 g of *trans*-ε-viniferin per kg d.w. of cane) and their market values, it is estimated [1] that it would be possible to obtain several thousand US dollars per hectare through the commercial use of these compounds. Zhang et al. [2] analysed 165 samples of canes from seven major wine regions in China using high-performance liquid chromatography, and prepared a very thorough economic calculation for obtaining *trans*-resveratrol from waste canes. Regarding extraction, Rayne et al. [1] recommended a mixture of alcohol and water (70–80% alcohol) for the quantitative extraction of *trans*-resveratrol and *trans*-ε-viniferin. Karacabey et al. [3] extracted from milled canes of the Pinot Noir variety using water alone and water with alcohol (7.4%, 15%, and 25%) in a pressure apparatus at temperatures of 85, 95, 105, 120, 140, and 160 °C. They found that the extraction yields were reduced at temperatures above 95 °C for both *trans*-resveratrol and *trans*-ε-viniferin. The maximum yield of *trans*-ε-viniferin amounted to 1.65 g/kg of lyophilized canes. It is difficult to compare the published data about the content of *trans*-resveratrol and *trans*-ε-viniferin in canes because various authors have studied different white and red varieties, including resistant and sensitive ones, and prepared samples of canes under different drying and storage conditions. Thus, the content of *trans*-resveratrol and *trans*-ε-viniferin varies in the range of 0.038–1.53 g/kg for *trans*-resveratrol and white varieties, 0.046–0.967 g/kg for *trans*-resveratrol and blue varieties, 0.62–1.92 g/kg for *trans*-ε-viniferin and white varieties, and 0.43–2.30 g/kg for *trans*-ε-viniferin and blue varieties [4]. Soural et al. [5] found as much as 6.03 g/kg of *trans*-resveratrol in the Cabernet Moravia variety. 

Pawlus et al. [6] studied the profile of nine stilbenoids in 16 grape varieties (*Vitis amurensis*, *Vitis arizonica*, *Vitis berlandieri*, *Vitis betulifolia*, *Vitis cinerea*, *Vitis* x *champini*, *Vitis* x *doaniana*, *Vitis labrusca*, *Vitis candicans* [syn. *Vitis mustangensis*], *Vitis riparia*, *Vitis rupestris*, *Vitis vinifera*, *Muscadinia rotundifolia*, *Vitis vinifera* x *Muscadinia rotundifolia*). Those authors isolated 15 stilbenoids, confirmed their structures by mass spectrometry and nuclear magnetic resonance spectrometry, and found that the greatest yield of *trans*-ε-viniferin was 5.74 g/kg in variety *Vitis riparia* Pull. Vergara et al. [7] studied the distribution of *trans*-resveratrol, *trans*-piceatannol, and *trans*-ε-viniferin in Chilean Pinot Noir and Gewürztraminer and found the highest concentration of *trans*-ε-viniferin of 0.87 g/kg in canes of Pinot Noir. They also found in this variety that—in contrast to other varieties—it is advantageous to keep the canes after trimming in the vineyard for a period of two months, during which the content of the *trans*-ε-viniferin practically doubled.

Guerrero et al. [8] described the cane stilbene composition of 22 grape cultivars. The authors chopped samples into small pieces, dried them at 40 °C, and performed the extraction using an acetone/H_2_O mixture at room temperature. The most abundant stilbene in all cultivars was *trans*-ε-viniferin, which reached concentrations ranging from 0.90 g/kg d.w. in Palomino fino to 2.81 g/kg d.w. in Gewürztraminer. Those authors also used principal component analysis (PCA) to better understand the differences between cultivars.

The great interest in *trans*-resveratrol in the past and in *trans*-ε-viniferin today is due to their biological properties. Certain of these properties are very promising from the viewpoint of human medicine. For example, *trans*-ε-viniferin glucoside is an attractive new candidate for protecting brain cells, and could therefore be useful in treating Alzheimer’s disease [9]. This probably relates to the finding that *trans*-(−)-ε-viniferin activity increases the activity of mitochondrial sirtuin 3 and protects cells in a model of Huntington’s disease, which is virtually untreatable [10]. *Trans*-ε-viniferin is active not only as a preventive agent against cancer, but also as a substance having direct cytotoxicity against selected cancer cell lines [11]. The results in a study published by Basri et al. [12] are very promising, and show that a combination of *trans*-ε-viniferin and vancomycin is active against methicillin-resistant *Staphylococcus aureus*—a bacterium responsible for difficult infections in humans and animals. Stilbenes from canes have also been studied as natural fungicides. Methanolic and ethanolic crude extracts from grape canes of Pinot Noir, Gamaret, and Divico varieties have exhibited significant antifungal activity against three major fungal pathogens affecting grapevines: *Plasmopara viticola, Erysiphe necator*, and *Botrytis cinerea* [13]. Stilbene complex mixtures from *Vitis vinifera* L. wastes (cane, wood, and root) have recently been used as a cheap source of bioactive compounds for the development of natural fungicides against, for example, *Plasmopara viticola* [14]. Because all the aforementioned and future studies have required and will require a starting material such as *trans*-ε-viniferin in far greater amounts, a successful search for sources of these substances and a description of their distribution in different grapevine varieties with regards to sampling procedure and storage conditions is highly desirable and promising. 

As is apparent from the overview presented above, it is necessary to thoroughly study the distribution of stilbenes in canes in relation not only to the variety and sampling points, but also to the variability in stilbenes content through time. Our work was therefore focused on studying and comparing stilbenes contents of seven varieties of grape canes in six different places in South Moravia, Czech Republic during a three-year study. To our knowledge, this is the first such comprehensive study of its kind.

## 2. Results

Seven varieties of *Vitis vinifera* L. from the Moravian wine region were included in the experiment, encompassing three blue grape varieties: Cabernet Moravia (CM; sampling points: 3, 5, 6), Blaufränkisch (Bl; sampling points: 3, 5, 6), and Laurot (La; sampling points: 1, 2, 6); and four white grape varieties: Chardonnay (Ch; sampling points: 3, 5, 6), Green Veltliner (GV; sampling points: 3, 5, 6), Hibernal (Hi; sampling points: 1, 4, 6), and Malverina (Ma; sampling points: 1, 4, 6). The varieties and six sampling sites are described in the Materials and Methods. In our study during 2014–2016, we focused on determining three dominant stilbenes in grape canes: *trans*-resveratrol, *trans*-ε-viniferin, and r2-viniferin.

**Figure 1:**
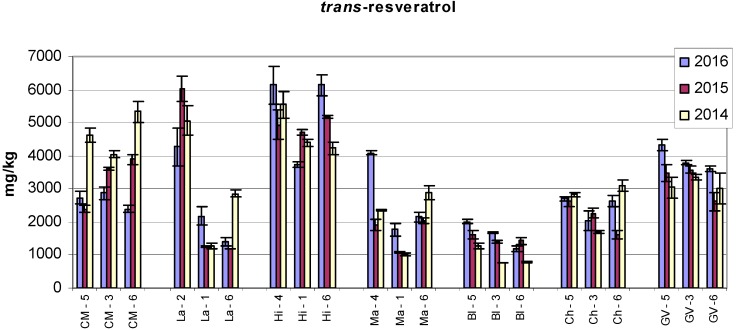
Contents of *trans*-resveratrol in grape canes during three years (contents in mg/kg dry weight). Example explanation of abbreviations: CM-5—“CM” is the variety, “5” is the sampling site (see Materials and Methods and Table 1). Bl: Blaufränkisch; Ch: Chardonnay; CM: Cabernet Moravia; GV: Green Veltliner; Hi: Hibernal; Ma: Malverina; La: Laurot.

**Figure 2:**
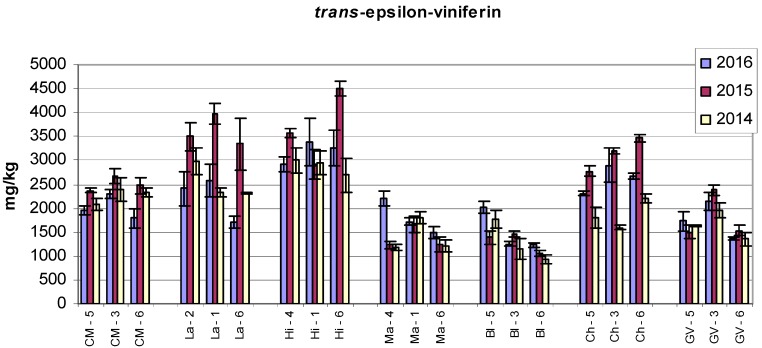
Contents of *trans*-ε-viniferin in grape canes during three years. Example explanation of abbreviations: CM-5—“CM” is the variety, “5” is the sampling site (see Materials and Methods and Table 1).

**Figure 3:**
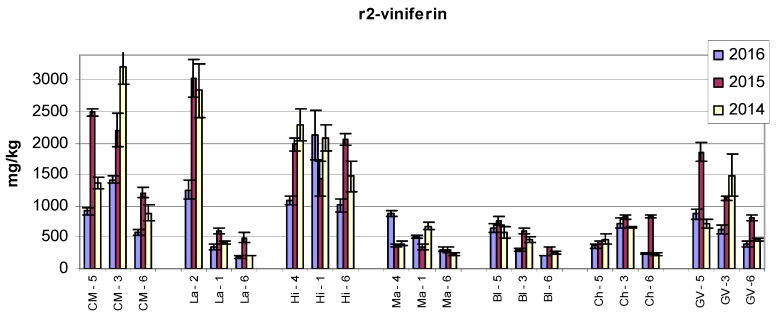
Contents of r2-viniferin in grape canes during three years. Example explanation of abbreviations: CM-5—“CM” is the variety, “5” is the sampling site (see Materials and Methods and Table 1).

Low values of *trans*-resveratrol were found in the Blaufränkisch (Bl) variety at all three sampling points and during all three observed years. The lowest values were recorded in 2014, and the difference was statistically significant as compared to 2015 and 2016 (Figure 1). It is interesting that the contents of the two other stilbenes—*trans*-ε-viniferin and r2-viniferin—in the Bl variety did not reach such high values (Figure 2 and Figure 3), but were relatively close to the values of the Malverina variety.

High values of all three stilbenes were found in the Hibernal variety. The amounts of *trans*-ε-viniferin in Laurot at all three sampling sites in 2015 were comparable to the values found in the Hibernal variety. The contents of r2-viniferin and *trans*-resveratrol in Laurot recorded at Sampling Site 2 were many times higher than those at Sampling Sites 1 and 6. A similar effect was also found in the Laurot variety for the contents of *trans*-resveratrol. Large differences in the contents of r2-viniferin at various sampling sites were also found in the Cabernet Moravia variety, and a significant difference was also determined between years.

Regarding r2-viniferin, it is interesting that in 2014, the lowest value (statistically significant, *p* < 0.05) was always recorded at Sampling Site 6 when comparing the same variety. With the exception of the Hibernal variety, the lowest value among different varieties in 2016 was also recorded at Sampling Site 6. Findings in the year 2015 were very diverse.

The highest amount of *trans*-resveratrol in the Cabernet Moravia variety was recorded at the sampling sites in 2014.

**Figure 4:**
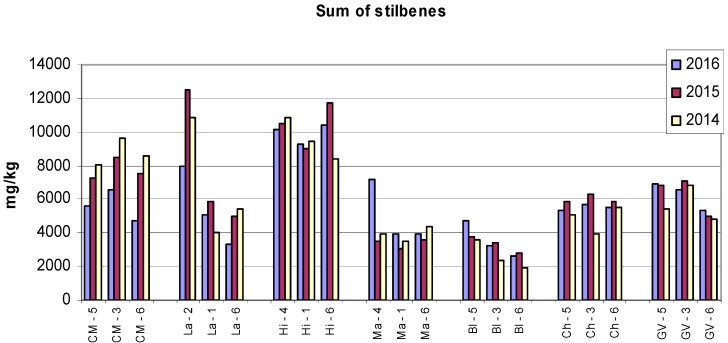
Total content of stilbenes in grape canes during three years. Example explanation of abbreviations: CM-5—“CM” is the variety, “5” is the sampling site (see Materials and Methods and Table 1).

Because the amounts of *trans*-resveratrol are approximately twice higher than those of *trans*-ε-viniferin and the amounts of r2-viniferin are smallest, the sums of all monitored stilbenes (Figure 4) essentially reflect the amounts of *trans*-resveratrol. The highest total content of stilbenes in the Cabernet Moravia variety at all sampling sites occurred in 2014. For the Laurot variety, Sampling Site 2 varied considerably from sites 1 and 6. For other varieties, with some exceptions, the differences between locations and years were small.

## 3. Discussion

**Figure 5:**
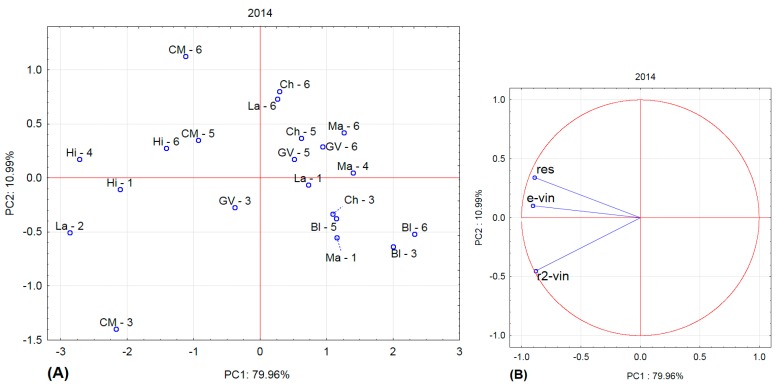
Principal component analysis for 2014. (**A**) projection of varieties and localities into component plane; (**B**) projection of component weights of stilbenes.

**Figure 6:**
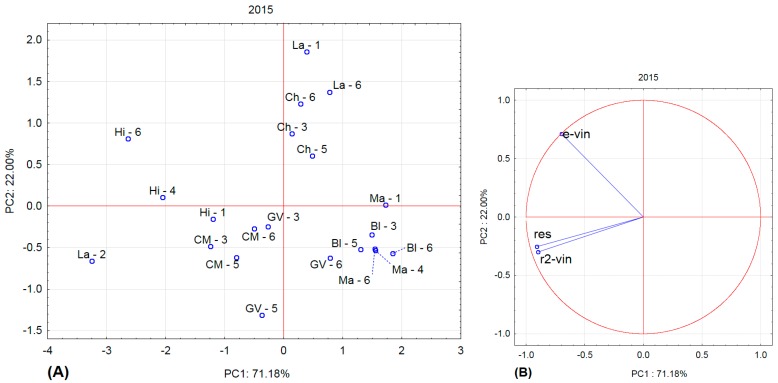
Principal component analysis for 2015. (**A**) projection of varieties and localities into component plane; (**B**) projection of component weights of stilbenes.

**Figure 7:**
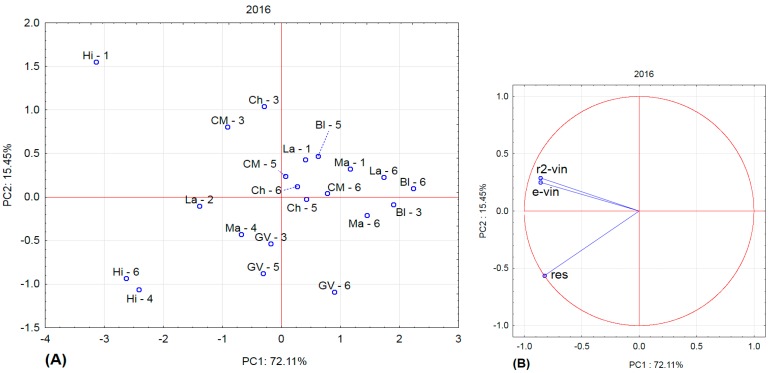
Principal component analysis for 2016. (**A**) projection of varieties and localities into component plane; (**B**) projection of component weights of stilbenes.

**Figure 8:**
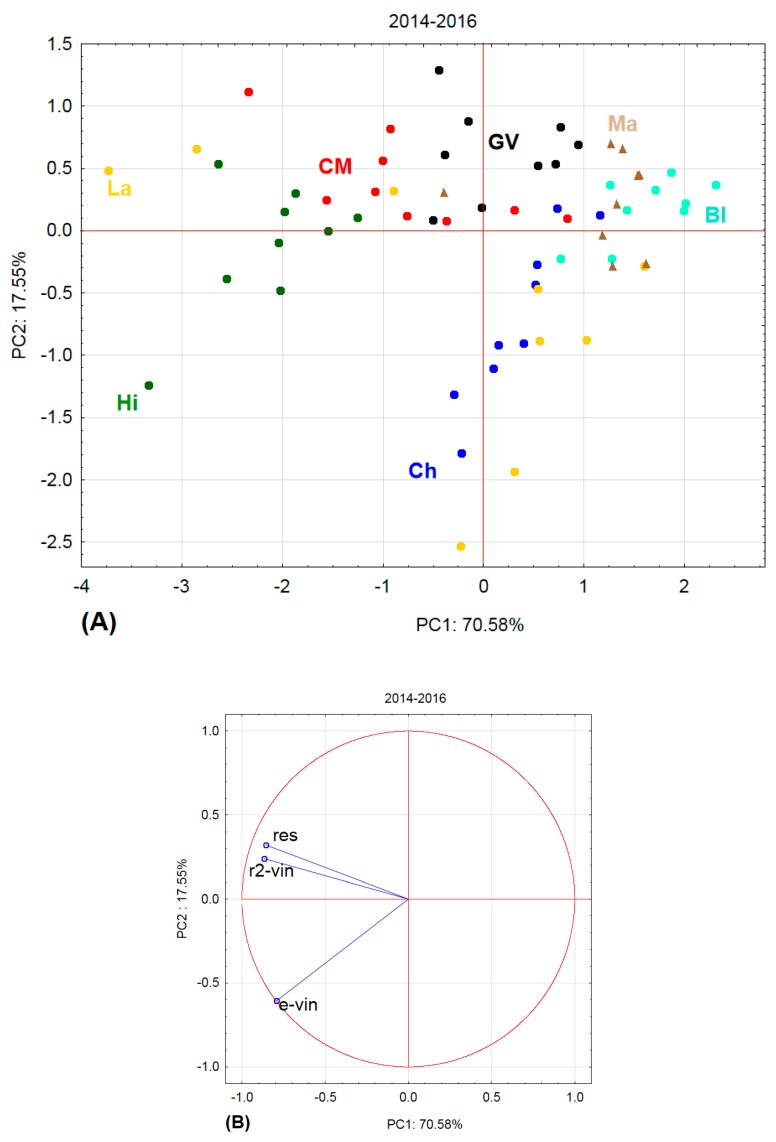
Principal component analysis for all three years. (**A**) projection of varieties and localities into component plane; (**B**) projection of component weights of stilbenes.

Figure 1, Figure 2, Figure 3 and Figure 4 show the amounts of stilbenes in the analysed varieties. To illustrate the relationships between varieties, locations, and years, we performed principal component analysis (PCA) using the Statistica 12 program. The results are presented in Figure 5, Figure 6, Figure 7 and Figure 8.

In 2014 (Figure 5), the Cabernet Moravia variety differed from other varieties and also differed by sampling site (CM-3 vs CM-5 and CM-6), as did Laurot (La-1 vs La-2 and La-6). Hibernal also differed from other varieties.

The Laurot variety differed again even in 2015, and differences in the contents of stilbenes in Laurot depended on location (La-1 vs La-2 and La-6). Hi-6 differed from the other Hibernal sites. The other varieties exhibited practically no dependence on the area of cultivation (i.e., sampling site), but a dependence on variety was obvious in the case of Chardonnay (3, 5, 6) vs Cabernet Moravia (3, 5, 6) vs Blaufränkisch (3, 5, 6). The variance in values for Green Veltliner and Malverina varieties was slightly larger.

In 2016, the Hibernal variety differed considerably from the others, and Sampling Sites 6 and 4 for Hibernal differed from Sampling Site 1.

The present results indicate that the difference is varietal for most varieties, regardless of the area of cultivation (sampling sites). Laurot and Hibernal varieties differ significantly, as in their cases we found a clear dependence on location (sampling sites).

The above results are complemented by PCA including all three years (Figure 8). For the sake of clarity, the various sampling sites are not indicated, but only the varieties according to colour. In the case of the varieties Malverina, Green Veltliner, and Blaufränkisch, the respective points are all located close together, indicating that the value of stilbenes in the respective canes does not depend on location (sampling sites) or year. In the case of Laurot, on the other hand, the points are spaced far apart and are located in three quadrants. Furthermore, it is obvious that the Hibernal variety differs from the other varieties. The spacing of the Chardonnay points is also interesting.

The issue of the diversity of the distribution of stilbenes obtained from grapevine canes is rather complex. As we have already pointed out in the article, the processes themselves (different storage and drying times) affect the contents of these substances. Our previous article [5] studied extraction methods for Cabernet Moravia, which affected yields. Because we have designed our sampling points so that at sampling point No. 6 (Znojmo—Oblekovice) we have all seven varieties, we added data for total rainfalls and sum of effective temperatures on this locality for 2014, 2015, and 2016 (see Table 2 Materials and Methods).

From the studied parameters, rainfall resulted higher in 2014. Due to the stress conditions which induce stilbene biosynthesis [15] (especially *trans*-resveratrol), the higher rainfall in 2014 might have increased the fungal pressure. This means that plants might have been stressed, and in this way the content of *trans*-resveratrol increased. It is obvious from Figure 1 that at sampling point No. 6 varieties CM, La, Ma, and Ch responded in the expected way, while varieties Hi, Bl, and GV responded in the opposite way.

Although the territory of the Moravian wine region is relatively small, we can still say that the contents of stilbenes in some varieties of vines was highly dependent on the year and location (sampling sites), but the effect was not found to be the same for all varieties. The reason may be that the varieties are not grown evenly across South Moravia. For all investigated varieties, the dominant stilbenes were *trans*-resveratrol, *trans*-ε-viniferin, and r2-viniferin.

To our knowledge, such an extensive set of data from a three-year study about the content of stilbenes in canes of different varieties of *Vitis vinifera* L. at various locations has not yet been published in the literature.

## 4. Materials and Methods

### 4.1. Varieties and Localities

Seven varieties of *Vitis vinifera* L. canes from the Moravian wine region were included in the experiment—three blue grape varieties: *Vitis vinifera* L. variety Cabernet Moravia (CM), *Vitis vinifera* variety Blaufränkisch (Bl), and Piwi variety Laurot (La); and four white grape varieties: *Vitis vinifera* L. variety Chardonnay (Ch), *Vitis vinifera* L. variety Green Veltliner (GV), Piwi variety Hibernal (Hi), and Piwi variety Malverina (Ma). The six sampling sites (1–6) are described in Table 1. Type of middle vine Rhine-Hesse training was used in all varieties. The canes from seven varieties were sampled at the end of February each year (2014, 2015, 2016).

### 4.2. Sample Preparation

Collected canes were dried in darkness for three months at room temperature. They were then ground using a laboratory grinder. Powdered grape canes were extracted for 165 min at 50 °C as described in our previous publication [5].

### 4.3. Chromatography Separation

The samples were separated using an HP 1050 high-performance liquid chromatography instrument (Hewlett–Packard, Palo Alto, CA, USA) on a 3 µm, 150 mm × 2 mm, Luna C18(2) column (Phenomenex, Torrance, CA, USA) with water-acetonitrile-o-phosphoric acid mobile phase. We used a G1315B diode array detector (DAD, Agilent, Prague, Czech Republic) and G1321A fluorescence detector (FLD, Agilent, Prague, Czech Republic). We followed the method detailed in our previous publication [5]. The content of *trans*-resveratrol was calculated according to a calibration curve for *trans*-resveratrol. The content of *trans*-ε-viniferin was calculated according to the calibration curve for *trans*-ε-viniferin, the content of r2-viniferin was calculated according to the calibration curve for *trans*-ε-viniferin in and the obtained value was multiplied by the ratio of MW of r2-viniferin/MW of *trans*-ε-viniferin. Data were recorded as mg/kg dry weight. The method was validated in terms of limits of detection and of linearity [16]. *Trans*-resveratrol (LOD 0.056 µg/mL, LOQ 0.187 µg/mL), *trans*-ε-viniferin (LOD 0.158 µg/mL, LOQ 0.525 µg/mL). 

### 4.4. Standards and Solvents

Standards were purchased from the following sources: *trans*-resveratrol (purity > 99%) from Sigma-Aldrich (Prague, Czech Republic), *trans*-epsilon viniferin from Sigma-Aldrich (Prague, Czech Republic), methanol, and acetonitrile from Merck (Prague, Czech Republic, LiChrosolv, gradient grade for LC), and *ortho*-phosphoric acid p.a. from Fluka (Prague, Czech Republic). 

## 5. Conclusions

The measurements of *trans*-resveratrol, *trans*-ε-viniferin, and r2-viniferin in studied cane extracts from six different places in South Moravia and from seven varieties of *Vitis vinifera* L. did not show the expected strong impact of locality. The largest difference was found in the variety Piwi Laurot at Sampling Site 2 for *trans*-resveratrol and r2-viniferin. A significant difference was also observed in the Cabernet Moravia variety at Sampling Sites 3 and 6 for r2-viniferin. From the viewpoint of producing extracts with high stilbenes content, the Hibernal variety is clearly the best. The mean amounts of the aforementioned stilbenes for this variety at all localities and for all three years was 4.99 g/kg for *trans*-resveratrol, 3.24 g/kg for *trans*-ε-viniferin, and 1.73 g/kg for r2-viniferin. 

## Figures and Tables

**Table 1:** Sampling sites of grape canes.

Sampling Site	Wine Area and Varieties	Wine Sub-Region
1	Lednice Hi, La, Ma	Mikulovská (GPS: 48°47′24.0″ N, 16°47′49.2″ E)
2	Hlohovec La	Mikulovská (GPS: 48°47′07.4″ N, 16°46′44.2″ E)
3	Velké Bílovice Bl, CM, GV, Ch	Velkopavlovická (GPS: 48°51′54.0″ N, 16°53′24.0″ E)
4	Rakvice Hi, Ma	Velkopavlovická (GPS: 48°51′46.5″ N, 16°48′00.3″ E)
5	Kostice Bl, CM, GV, Ch	Slovácká (GPS: 48°44′28.8″ N, 16°58′28.5″ E)
6	Znojmo-Oblekovice Bl, CM, GV, Hi, Ch, La, Ma	Znojemská (GPS: 48°49′26.4″ N, 16°05′30.8″ E)

**Table 2:** Wine Area: Znojmo—Oblekovice (sampling site 6).

Year	2016	2015	2014
Rainfall total (mm)	428	359	545
Sum of effective temperatures (°C)	1450	1470	1337
